# Comparing the efficacy of glucocorticoids and anti-VEGF in treating diabetic macular edema: systematic review and comprehensive analysis

**DOI:** 10.3389/fendo.2024.1342530

**Published:** 2024-03-22

**Authors:** Zhi’ang Cheng, Xiaoyong Liu

**Affiliations:** ^1^ Department of Ophthalmology, The First Affiliated Hospital of Jinan University, Guangzhou, Guangdong, China; ^2^ Department of Ophthalmology, The Affiliated Shunde Hospital of Jinan University, Foshan, Guangdong, China

**Keywords:** diabetic macular edema, network meta-analysis, anti-vascular endothelial growth factor, triamcinolone acetonide, dexamethasone, best corrected visual acuity, central macular thickness

## Abstract

**Introduction:**

The aim of this study was to better understand the efficacy of various drugs, such as glucocorticoids and anti-vascular endothelial growth factors (VEGF), in the treatment of diabetic macular edema (DME), and to evaluate various clinical treatment regimens consisting of different therapeutic measures.

**Methods:**

This study included randomized controlled trials up to February 2023 comparing the efficacy of corticosteroid-related therapy and anti-VEGF therapy. PubMed, the Cochrane Library, and Embase were searched, and the quality of the studies was carefully assessed. Finally, 39 studies were included.

**Results:**

Results at 3-month followup showed that intravitreal injection of bevacizumab (IVB) + triamcinolone acetonide (TA) was the most beneficial in improving best-corrected visual acuity and reducing the thickness of macular edema in the center of the retina in patients with DME. Results at 6-month follow-up showed that intravitreal dexamethasone (DEX) was the most effective in improving patients’ bestcorrected visual acuity and reducing the thickness of central macular edema.

**Discussion:**

Overall, IVB+TA was beneficial in improving best-corrected visual acuity and reducing central macular edema thickness over a 3-month follow-up period, while DEX implants had a better therapeutic effect than anti-VEGF agents at 6 months, especially the patients with severe macular edema and visual acuity impaired.

**Systematic review registration:**

https://www.crd.york.ac.uk/PROSPERO/display_record.php?RecordID=397100, identifier CRD42023397100.

## 1 Introduction

Diabetes mellitus is one of the common chronic diseases that affect the quality of life of middle-aged and older people. The high glucose state of diabetes can damage systemic blood vessels. When it acts on microvessels, it is mainly manifested as diabetic retinopathy, which is the main cause of newly diagnosed blindness in old people (1, 2). The number of people with visual impairment caused by diabetes is increasing year by year. It is estimated that by 2030, the number of patients with diabetic retinopathy worldwide will increase from 103 million in 2020 to 130 million (3). DME can occur at any stage of the non-proliferative and proliferative phases of diabetic retinopathy, showing progressive aggravation. Therefore, the treatment of diabetic retinopathy aims to delay the disease process, improve the existing visual quality of patients, and ameliorate the quality of life-related to vision.

There are two main mechanisms for the development of DME. First, the state of hyperglycemia promotes retinal vascular degeneration, ischemia and hypoxia in the posterior pole of the retina, and the local accumulation of large amounts of vascular endothelial growth factor (VEGF), resulting in the formation of large numbers of new vessels with high brittleness. Second, inflammation causes an increase in vascular permeability, leading to fluid exudation, which accumulates in the layers of ganglion cells in the macula of retina, causing macular edema (2). Clinically known as DME, it is one of the important causes of visual loss in patients with advanced diabetes. In view of the above-mentioned mechanism, in addition to surgical treatment such as laser photocoagulation and vitrectomy, the most commonly used drug therapy is intravitreal injection of vascular endothelial growth factor antibodies (such as abscisic, bevacizumab, conbercept, etc.) or glucocorticoid (such as triamcinolone acetonide, dexamethasone intravitreal implants, etc.) (4). However, there is no consensus on the efficacy and safety of various therapeutic regimens.

To date, there have been relevant network meta-analyses comparing the efficacy and safety of anti-VEGF drugs in diabetic macular edema, but they did not include glucocorticoids. Given the limitations of head-to-head RCTs or traditional meta-analyses, in order to obtain evidence for direct and indirect comparisons and cross-sectional analysis of the efficacy of various interventions in diabetic macular edema, thus providing the best clinical evidence, we conducted a network meta-analysis to comprehensively compare the efficacy of various drugs such as glucocorticoids and anti-VEGF in diabetic macular edema, and to evaluate clinical treatment regimens composed of different treatment measures.

## 2 Methods

This study followed the PRISMA statement for network meta-analysis (5), and has been previously registered with PROSPERO (Registration Code: CRD42023397100).

### 2.1 Literature screening

**Figure 1:**
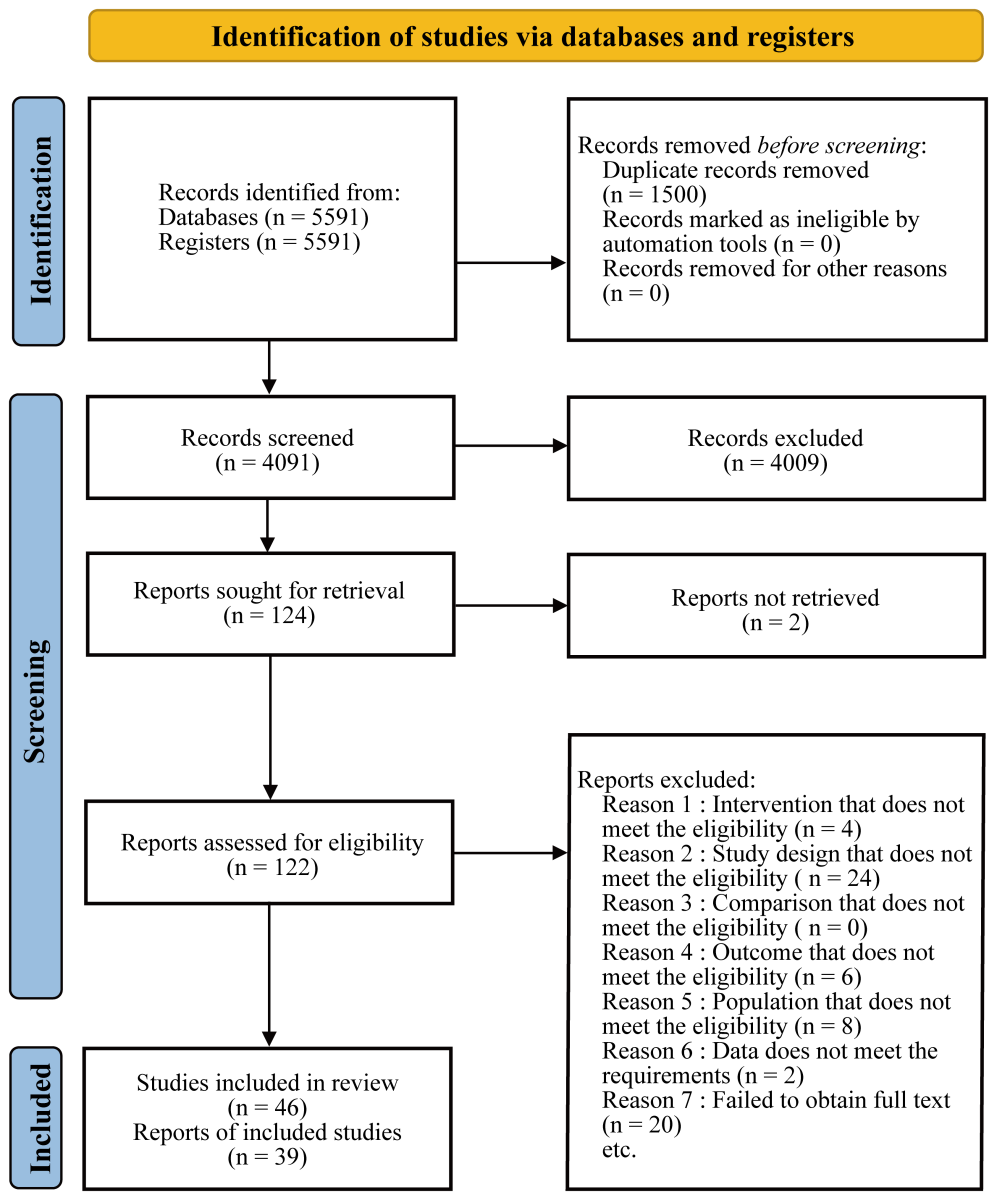
PRISMA flow chart.

We searched PubMed, the Cochrane Library, and Embase for published articles from database inception to February 2023 without restrictions on time, date, language, and type of article. The search strategy and all data are detailed in the attachment. Two researchers conducted preliminary screening based on the title and abstract of the search results, and then obtained the full text for more detailed data screening. Disagreements were resolved through full discussion, and consultation with a third researcher if necessary. The process of this network meta-analysis was presented in the **Figure 1**.

The inclusion and exclusion criteria for screening were as follows, and the inclusion criteria were formulated according to the PICOS principle:

Participants: Patients with DME (more than 3 years); Age>40 years; Male or female; best-corrected visual acuity lower than 0.8; central macular thickness at least 320μm or more; no prior history of intravitreal or peribulbar injections of drugs or vitrectomy. The duration of patients with diabetic macular edema exceeds 1year; all patients involved in the center of the macula. We classify diabetic macular edema according to the thickness of the central macular area: 1) mild diabetic macular edema with the thickness of 320 to 450μm; 2) severe macular edema with the thickness >450μm (450μm not included).

Intervention measures: all intervention measures related to glucocorticoids such as DEX and TA; To provide more information for indirect comparison, laser photocoagulation combined with glucocorticoids and intravitreal injection of anti-VEGF drugs combined with glucocorticoids were also included in the study; There were no special requirements for treatment dose, frequency, time, mode of administration, treatment course, etc.

Comparison: laser photocoagulation or placebo group (including sham injection or sham laser).

Outcome measures: The primary outcome measures were the best corrected visual acuity and central macular thickness.

Study type: All included studies were RCTs.

Exclusion criteria were as follows:

All articles in languages other than English and Chinese were excluded; Articles with only abstract or preface available but missing full text were excluded; Research articles that had not been peer-reviewed were excluded; Non-RCTs were excluded, including literature review, systematic review, case report and retrospective study; When multiple study results were published for studies with long follow-up time, only the results with the longest follow-up time or the largest sample size were generally included; Studies that included patients with diabetic retinopathy, with or without macular edema, in the same trial should be excluded.

### 2.2 Data extraction and quality assessment

Two investigators jointly extracted the following information from the included studies: Study author and publication year, basic information of patients (such as age, gender, nationality, diagnosis, clinical stage of disease, severity of disease, study sample size, baseline visual acuity, central macular thickness, baseline intraocular pressure, etc.), information on intervention measures (including drug dose, injection frequency), information on outcome indicators (such as follow-up time, outcome data). The primary outcome indicator was the change in the best corrected visual acuity. Secondary outcome measures were changes in retinal anatomy such as central macular thickness. All outcome measures were recorded at 3- and 6-month follow-up. All disagreements during data extraction were resolved by thorough discussion.

We evaluated the quality of evidence for all included RCTs using the built-in ROB tool (version 1.0) in Review Manager (version 5.4.1), including the generation way of random sequences, allocation concealment, blinding of subjects, blinding of outcome measures, incomplete data on outcome measures, selective reporting, and other biases. All disagreements were resolved by thorough discussion.

### 2.3 Data analysis

Before the analysis, we already know that there were two forms of BCVA report in the existing research, namely LogMar and Letters. For the convenience of analysis, we use a formula to convert all Letters into LogMar. The formula was as follows:


LogMar=1.7−(The number of sighting targets correctly identified × 0.02)

We performed a Bayesian network meta-analysis using the GeMTC (version 1.0 - 1) package in R Studio 4.1.3. The two outcome measures included in the study, BCVA and CMT, were continuous variables and were therefore represented by means and standard deviations. The implementation of network meta-analysis needs to meet three basic assumptions, namely transitivity assumption, homogeneity assumption and consistency assumption. At present, there was no accepted statistical test method for transitivity hypothesis. In this study, the similarity of population characteristics included in the study was judged by using the data in the basic information table. If the population was generally similar, it was considered that the transitivity hypothesis is satisfied. Heterogeneity analysis was performed using the mtc.anohe function in the GeMTC package, and when the overall I^2^ was less than 50%, the heterogeneity of each included study within the same comparison was considered acceptable and the homogeneity assumption was met. The inconsistency between the direct comparison and the indirect comparison was checked using the mtc. nodessplit function in the GeMTC package by the node splitting method. When p>0.05, it was considered that there is no inconsistency between direct comparison and indirect comparison, and the consistency assumption is satisfied. In the network meta-analysis, although strict inclusion and exclusion criteria were established and heterogeneity analysis was passed, there were still inherent differences between studies that might affect the analysis results, so the random-effects model was directly used for analysis in this study.

After completing the test of the above assumptions, 1) the network was constructed with the interventions as nodes, and the lines between the nodes indicated the existence of direct comparison between the two interventions; 2) a forest plot of relative effects was drawn with the placebo group as a control by comparing the other interventions with placebo; 3) a league table was generated with the results of the analysis of relative effects, and the values in each cell indicated the difference in means between the intervention represented by its column and its row; 4) the cumulative probability ranking chart was analyzed, and all the cumulative ranking probabilities were estimated and reported as the surface area under the cumulative ranking curve (SUCRA).

## 3 Results

### 3.1 Basic characteristics of included studies

A total of 39 studies were included, including 5823 eyes of patients with diabetic macular edema and 10 interventions to treat the disease. Among them, TA was used in 19 studies (983 eyes), IVB in 12 studies (676 eyes), LP in 20 studies (2097 eyes), Placebo in 8 studies (2375 eyes), IVA in 3 studies (173 eyes), TA+LP in 7 studies (928 eyes), DEX+LP in 4 studies (487 eyes), DEX in 8 studies (2868 eyes), IVB+TA in 8 studies (508 eyes) and IVR in 1 study (363 eyes). The visual acuity fluctuation of the included patients before intervention was within the range of 0.1-0.8, and the macular thickness in the center of retina was within the range of 320-600 mm. The population characteristics of the included patients were highly similar, which could meet the transitivity hypothesis of network meta-analysis. The basic characteristics of the included studies are provided in **Table 1** and **Supplementary Table S1**.

**Table 1:** Study characteristics of randomized controlled trials included in the network meta-analysis.

Study	Region	No.	Eyes	Age	Gender (female, male)	BCVA	CMT	Intervention	Control
Audren 2006 (6)	France	17	34	60.1,9.0	NA	0.72,0.28	528.70,155.78	TA	Placebo
Aydin 2009 (7)	Turkey	30	49	60.3,8.4	17,13	0.77,0.23	NA	TA	TA+LP
Azad 2012 (8)	India	40	40	52.4,5.9	15,25	0.85,0.11	425.15,102.41	TA	IVB
Bhayana 2015 (9)	India	30	30	57.7,7.4	18,12	0.70,0.41	444.00,74.86	TA	IVB
Boyer 2014 (10)	Multicenter	1048	1048	62.4,9.0	412,636	0.58,0.19	NA	DEX	Placebo
Callanan 2013 (11)	Multicenter	253	253	61.5,10.2	125,128	0.56,0.19	456.51,131.26	DEX+LP	LP
Callanan 2017 (12)	Multicenter	363	363	63.6,9.7	135,228	0.49,0.19	468.01,137.86	DEX	IVR
Comet 2021 (13)	France	41	41	67.9,8.6	18,23	0.53,0.29	466.94,129.64	DEX	IVA
Danis 2016 (14)	Multicenter	1034	1034	62.4,9.0	412,636	0.58,0.19	463.56,150.08	DEX	Placebo
Dehghan 2008 (15)	Iran	61	88	61.6,8.8	27,34	0.93,0.33	393.00,157.59	TA	Placebo
Elman 2010 (16)	American	NA	479	62.6,9.8	NA	0.39,0.24	394.19,118.71	TA+LP	LP
Emily 2007	American	NA	82	58.6,10.4	35,47	0.10,0.10	328.28,68.58	TA	LP
Emily 2007’	American	NA	85	61.0,11.0	29,56	0.12,0.12	326.24,63.51	TA+LP	LP
Faghihi 2008 (17)	Iran	NA	83	57.5,6.6	41,42	0.73,0.32	371.31,136.16	IVB+TA	IVB
Fazel 2023 (18)	Iran	NA	58	62.6,5.9	39,19	0.79,0.30	521.79,132.62	IVB+TA	IVB
Gao 2022	China	36	36	67.7,8.9	20,16	0.60,0.22	500.78,72.47	DEX+LP	LP
Gil 2011 (19)	Brazil	14	21	59.3,6.0	10,4	0.88,0.30	405.37,33.52	TA	LP
Gillies 2010 (20)	Australia	54	84	66.2,9.2	36,48	0.59,0.24	479.75,139.24	TA+LP	LP
Gillies 2014 (21)	Australia	61	88	61.8,9.7	36,52	0.58,0.24	487.99,119.67	DEX	IVB
Heng 2016 (22)	UK	80	80	63.4,11.9	14,66	0.37,0.24	455.50,114.71	DEX+LP	LP
Isaac 2012 (23)	Brazil	11	22	64.6,9.8	5,6	0.72,0.30	490.50,102.03	TA	IVB
Jonas 2004 (24)	Germany	25	50	66.6,8.3	NA	0.78,0.30	NA	TA	Placebo
Kriechbaum 2014 (25)	Austria	30	30	59.0,11.0	18,12	0.31,0.20	497.50,110.34	TA	IVB
Lam 2007 (26)	HongKong	73	73	65.5,9.3	37,36	0.66,0.35	404.23,105.14	TA+LP	LP
Lam 2007’ (26)	HongKong	75	75	66.7,9.0	41,34	0.68,0.35	390.57,95.06	TA	LP
Larsson 2009 (27)	Australia	16	32	62.0,8.5	5,11	0.44,0.24	NA	TA	Placebo
Lee 2009 (28)	Korea	54	60	61.6,11.0	32,22	0.57,0.31	506.05,41.80	TA+LP	LP
Li 2014 (29)	China	64	64	53.3,13.8	26,38	NA	542.80,122.14	TA+LP	LP
Maia Jr 2009 (30)	Brazil	22	44	61.9,5.3	12,10	0.41,0.17	345.86,82.22	TA	LP
Marey 2011 (31)	Egypt	60	60	57.7,7.3	23,37	0.73,0.35	485.00,148.60	TA	IVB+TA
Marey 2011’ (31)	Egypt	60	60	57.6,7.2	26,34	0.70,0.31	468.68,136.28	TA	IVB
Marey 2011’’ (31)	Egypt	60	60	NA	NA	0.69,0.32	461.38,139.20	IVB+TA	IVB
Massin 2004 (32)	France	12	24	59.0,9.2	7,5	0.70,0.27	492.00,115.90	TA	Placebo
Meyer 2022 (33)	Australia	52	52	60.2,6.9	35,17	0.44,0.32	402.15, 120.74	DEX	IVB
Ockrim 2008 (34)	England	43	88	63.6,9.0	28,60	0.62,0.27	411.86,130.31	TA	LP
Ogura 2019 (35)	Japan	89	89	65.2,9.0	36,53	0.55,0.20	479.11,120.95	TA	LP
Ozsaygili 2020 (36)	Turkey	62	98	65.7,5.6	27,35	0.76,0.08	594.60,117.20	DEX	IVA
Soheilian 2007 (37)	Iran	NA	66	63.4,6.3	34,32	0.66,0.35	345.00,138.95	IVB+TA	LP
Soheilian 2009 (38)	Iran	NA	100	61.4,6.4	49,51	0.72,0.28	350.00,142.69	IVB+TA	IVB
Soheilian 2009’ (38)	Iran	NA	100	61.7,6.1	44,56	0.64,0.28	329.50,130.62	IVB+TA	LP
Stefansson 2023 (39)	Multicenter	144	144	64.4,9.7	52,92	0.44,0.19	464.52,133.62	DEX	LP
Sutter 2004 (40)	Australia	40	65	64.5,2.3	18,22	0.49,0.25	441.03,113.27	TA	Placebo
Wei 2021 (41)	China,Philippines	272	272	59.3,7.9	133,139	0.59,0.22	486.95,157.62	DEX+LP	LP
Yaseri 2014 (42)	Iran	19	19	NA	NA	0.74,0.33	340.84,154.39	IVB+TA	IVB
Yaseri 2014’ (42)	Iran	22	22	NA	NA	0.55,0.28	314.50,128.70	IVB+TA	LP

*BCVA, best-corrected visual acuity; CMT, central macroscopic thickness.

*TA, intravitreal triamcinolone; IVB, intravitreal bevacizumab; LP, laser, macroscopic laser, grid laser and focal/grid laser; TA+LP, intrareal triamcinolone combined with laser; DEX, intravitreal dexamethasone; IVB+TA, intravitreal bevacizumab combined with triamcinolone; DEX+LP, intrareal dexamethasone combined with laser; IVR, intravitreal ranibizumab; IVA, intravitreal affiliation.

### 3.2 Evaluation of evidence quality and data extraction

**Figure 2:**
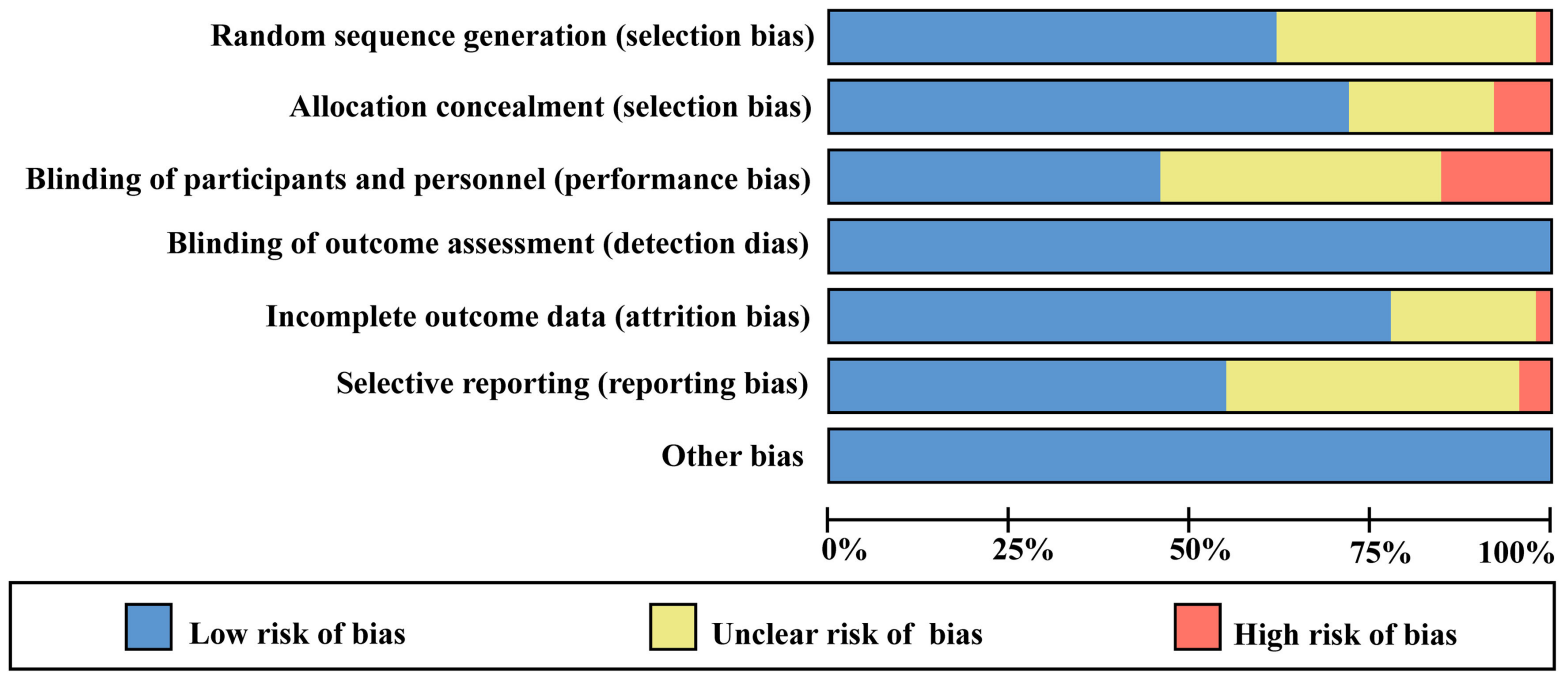
Risk of bias graph: review authors’ judgments about each risk of bias item presented as percentages across all included studies.

**Figure 3:**
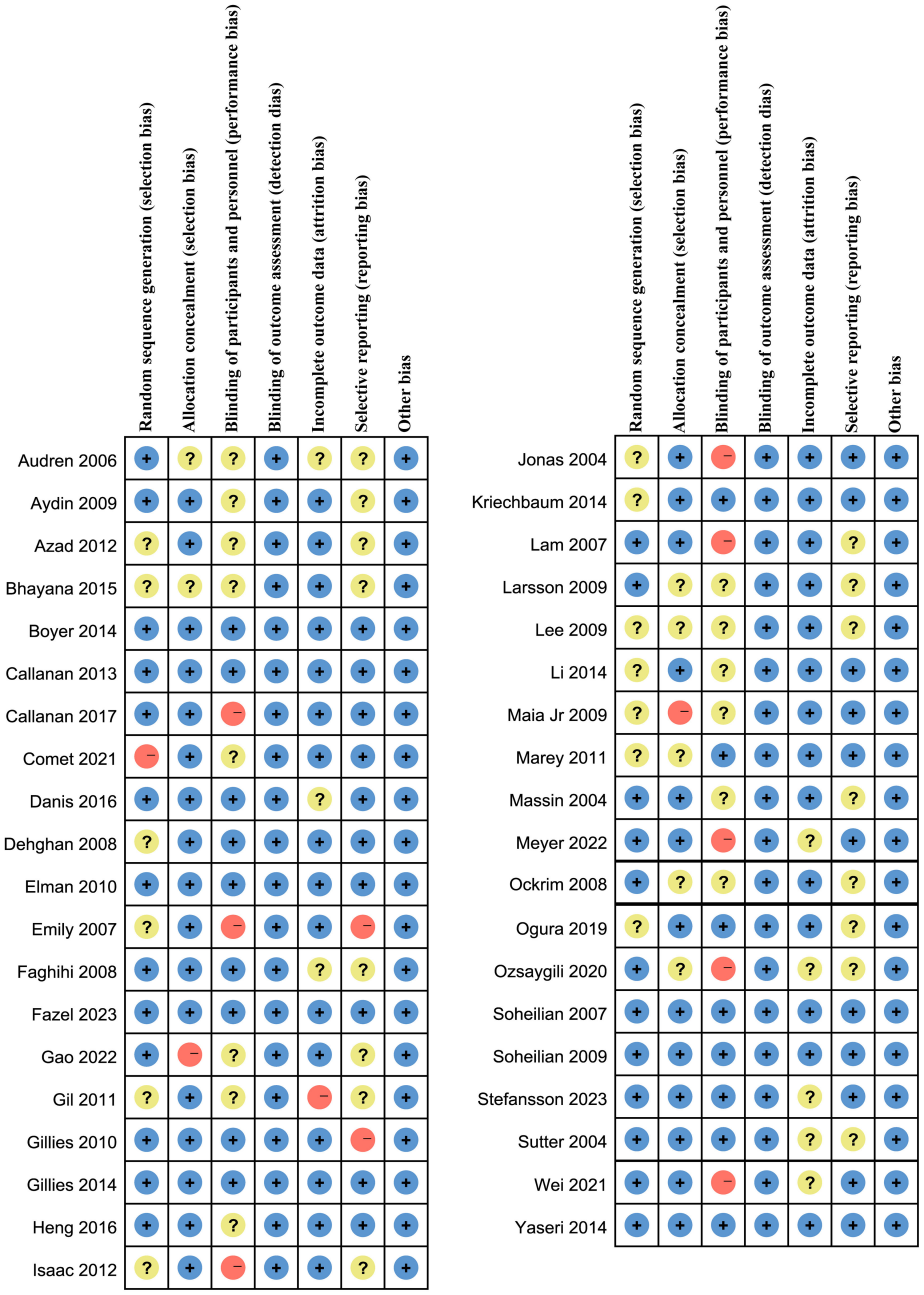
Assessment of the risk of bias in included studies. Risk of bias summary: review authors’ judgments about each risk of bias items for each included study. +: Low risk of bias; -: High risk of bias; ?: Clear risk of bias.

As shown in **Figures 2** and **3**, 14 studies did not describe how to randomly allocate patients. Although these studies claimed to be randomized design, they did not elaborate on what kind of random allocation method was used. Nine studies were not assigned for concealment, one of which were open label. Seven studies had a high-risk bias, and 14 studies did not have a double-blinded or triple-blinded design for patients and researchers, with an open label design. For all included studies, no bias was found in the outcome evaluation blind method. Eight studies had incomplete outcome indicators, and one of them had a high-risk bias. Selective reporting bias occurred in 2 studies, of which 17 had medium-risk biases. For the evaluation of bias items not mentioned above, it can be summarized as other biases. No serious defects such as unreasonable design of patient inclusion criteria were found, such as patients receiving laser or other treatment prior to inclusion in this study, which may affect the results of clinical trials and cause a certain degree of bias risk (6–49).

### 3.3 Inconsistency and heterogeneity analysis of network model

The heterogeneity and inconsistency of the network meta-analysis were tested. In the inconsistency test, the P value of the inconsistency test of all studies was greater than 0.05, indicating that there is no significant inconsistency between the direct comparison and the indirect comparison of the included studies, and the consistency hypothesis is satisfied. The analysis results were shown in Annex 1. According to heterogeneity analysis, the I^2^ value of most studies was less than 50%. Although there was heterogeneity in a few studies, it had little impact on the homogeneity hypothesis of this study. Overall, there was no significant heterogeneity, indicating that our network meta-analysis met the homogeneity hypothesis. The analysis results were shown in Annex 2.

### 3.4 Mean change in best-corrected visual acuity at 3 months

**Figure 4:**
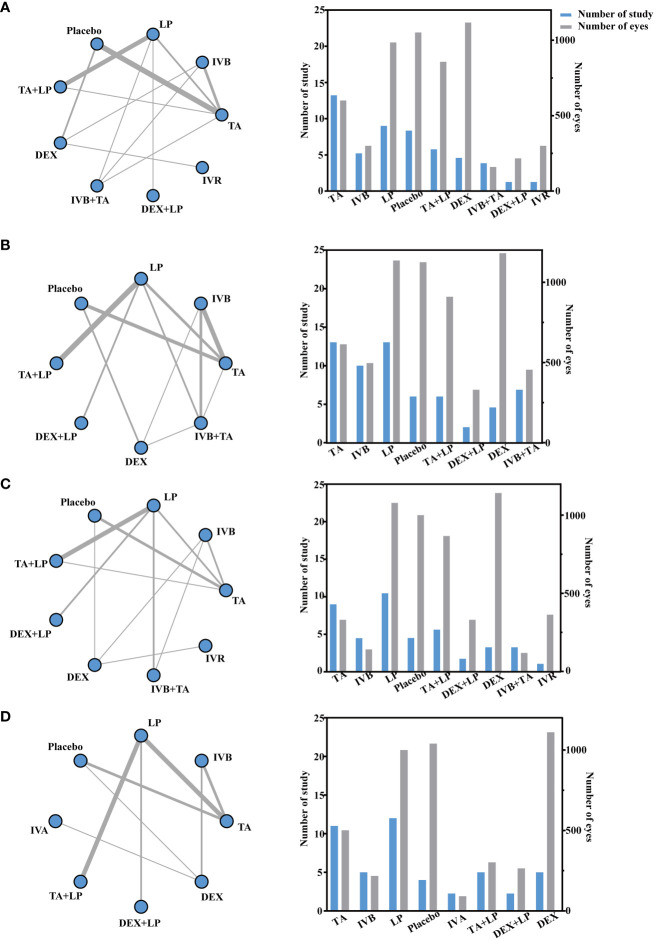
Network geometry for BCVA mean change from baseline. All populations at 3-months follow-up [**(A)** 25 trials] and 6-months follow-up [**(C)** 21 trials]. Network geometry for CMT mean change from baseline. All populations at 3-months follow-up [**(B)** 30 trials] and 6-months follow-up [**(D)** 23 trials]. BCVA, best-corrected visual acuity; CMT, central macular thickness. TA, intravitreal triamcinolone; IVB, intravitreal bevacizumab; LP, laser, macroscopic laser, grid laser and focal/grid laser; IVA, intravitreal affiliation; TA+LP, intrareal triamcinolone combined with laser; DEX, intravitreal dexamethasone; IVB+TA, intravitreal bevacizumab combined with triamcinolone; DEX+LP, intrareal dexamethasone combined with laser; IVR, intravitreal ranibizumab. Direct comparisons are represented by the black lines connecting the different interventions. Line width is proportional to the number of trials including every pair of interventions.

**Figure 5:**
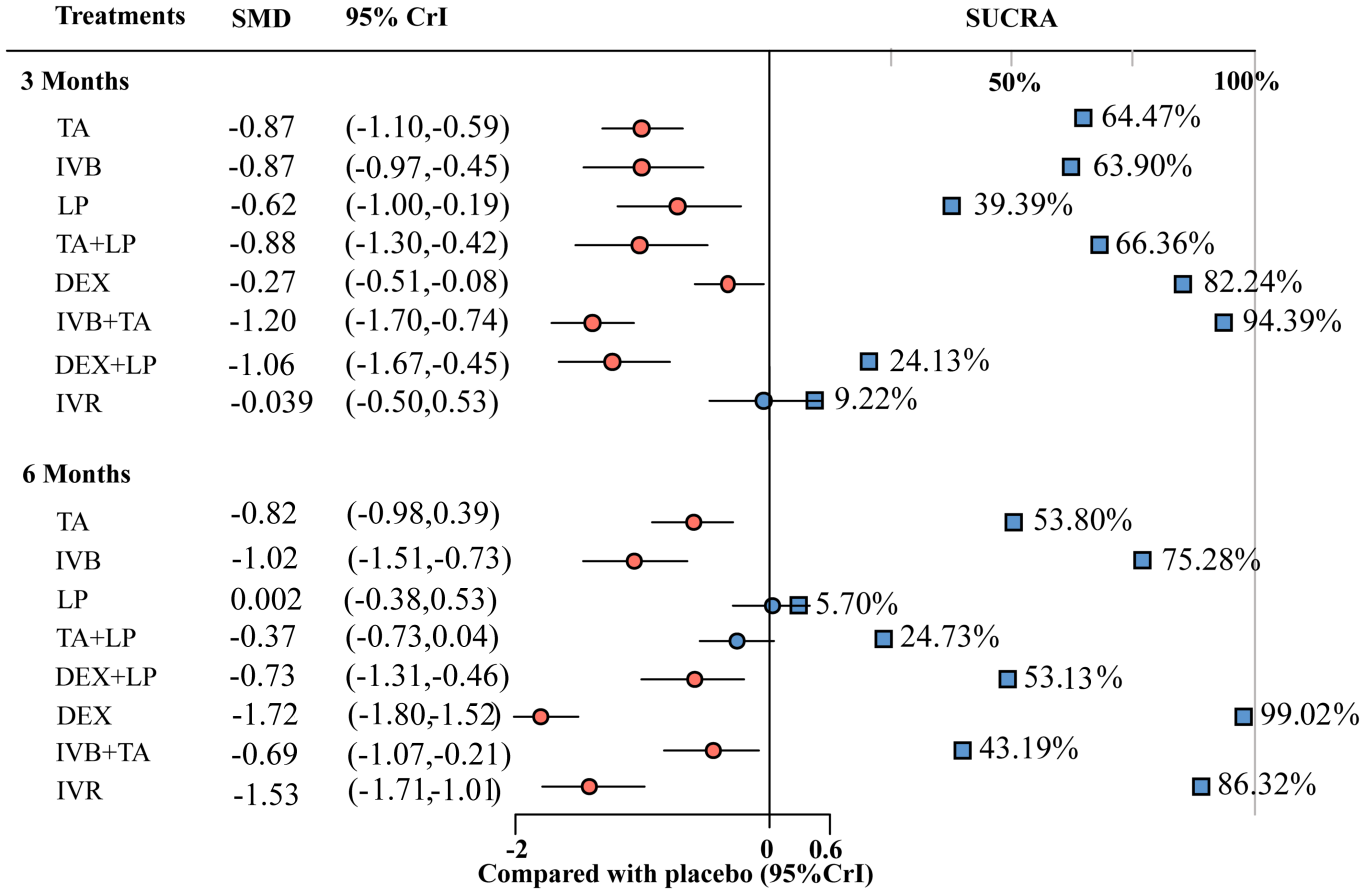
Forest plots for BCVA at 3 months and 6 months. TA, intravitreal triamcinolone; IVB, intravitreal bevacizumab; LP, laser, macroscopic laser, grid laser and focal/grid laser; TA+LP, intrareal triamcinolone combined with laser; DEX, intravitreal dexamethasone; IVB+TA, intravitreal bevacizumab combined with triamcinolone; DEX+LP, intrareal dexamethasone combined with laser; IVR, intravitreal ranibizumab.

A total of 27 studies involving 3770 eyes were included in this study, with a total of 9 interventions including TA, IVB, LP, Placebo, TA + LP, DEX, IVB + TA, DEX + LP and IVR. A network meta-analysis was performed and a network plot was constructed (**Figure 4A**). In the relative effect forest plot, IVB + TA had the best effect on improving visual acuity. TA + LP, IVB and TA had similar effects on improving visual acuity, and Placebo was the worst. The values for each intervention compared with placebo were shown in the league table (**Table 2**). The results showed that there were significant differences in the improvement of the best corrected visual acuity among the interventions. The SUCRA values of each intervention were shown in the **Table 3**, and IVB + TA has a maximum value of 94.39% compared with other interventions. According to the rank chart and league table, IVB + TA was the best treatment plan to improve the best corrected visual acuity at 3 months (**Figure 5**).

**Table 2:** Network meta-analysis results in BCVA and CMT at 3 months (lower part) and 6 months (upper part).

**TA**	-0.36 (-0.6, -0.12)	-0.14 (-0.37, 0.09)	-0.78 (-1.05, -0.51)	**0.34** **(0.05, 0.65)**	**0.54** **(0.15, 0.93)**	**0.62** **(0.35, 0.87)**	**0.54** **(0.15, 0.93)**	-	
	0.07 (-0.39, 0.55)	-0.54 (-1, -0.16)	-0.46 (-0.92, -0.02)	-0.15 (-0.75, 0.39)	**1.60** **(1.03, 2.24)**	-	0.34 (-0.42, 1.01)	0.55 (-0.43, 1.61)	
-0.01 (-0.35, 0.33)	**IVB**	0.21 (-0.08, 0.51)	-0.42 (-0.76, -0.08)	**0.69** **(0.36, 1.06)**	**0.90** **(0.50, 1.33)**	**0.97** **(0.72, 1.23)**	**0.91** **(0.50, 1.33)**	-	
0.41 (-0.06, 0.73)		-0.61 (-1.29, -0.02)	-0.53 (-1.12, 0.04)	-0.22 (-1.01, 0.48)	**1.52** **(1.01, 2.09)**	-	0.27 (-0.65, 1.07)	0.48 (-0.48, 1.5)	
-0.25 (-0.58, 0.07)	-0.25 (-0.68, 0.21)	**LP**	-0.63 (-0.98, -0.29)	**0.49** **(0.32, 0.68)**	**0.69** **(0.4, 0.98)**	**0.76** **(0.49, 1.02)**	**0.69** **(0.37, 0.98)**	-	
**-0.76** **(-1, -0.51)**	**-1.17** **(-1.54, -0.75)**		0.07 (-0.5, 0.73)	**0.39** **(0.02, 0.77)**	**2.13** **(1.47, 2.95)**	-	**0.87** **(0.30, 1.44)**	**1.09** **(0.06, 2.28)**	
**-0.87** **(-1.14, -0.59)**	**-0.87** **(-1.25, -0.45)**	**-0.63** **(-1.02, -0.19)**	**Placebo**	**1.21** **(0.73, 1.52)**	**1.32** **(0.88, 1.78)**	**1.39** **(1.05, 1.75)**	**1.32** **(0.88, 1.78)**	-	
**-0.74** **(-1.02, -0.47)**	**-1.14** **(-1.49, -0.78)**	0.02 (-0.34, 0.38)		0.32 (-0.45, 1.01)	**2.07** **(1.52, 2.70)**	-	0.80 (-0.09, 1.6)	**1.01** **(0.05, 2.08)**	
0.01 (-0.37, 0.37)	0.01 (-0.45, 0.49)	0.26 (0.02, 0.48)	0.88 (0.41, 1.31)	**TA+LP**	0.21 (-0.14, 0.53)	0.28 (-0.06, 0.59)	0.21 (-0.14, 0.53)	-	
**-0.43** **(-0.73, -0.16)**	0.64 (-0.41,1.24)	0.33 (0.18, 0.47)	0.31 (-0.08, 0.69)		**1.74** **(0.98, 2.65)**	-	0.48 (-0.21, 1.17)	0.69 (-0.4, 1.96)	
**-0.59** **(-1.01, -0.26)**	**-0.60** **(-1.05, -0.18)**	-0.37 (-0.86, 0.1)	0.27 (0.09, 0.51)	0.62 (0.12,1.14)	**DEX**	**0.67** **(0.29, 1.04)**	**0.58** **(0.12, 1.07)**	-	
-0.01 (-0.4, 0.36)	**-0.42** **(-0.89, -0.07)**	0.75 (0.48, 1.02)	0.73 (0.27, 1.18)	0.42 (-0.12, 0.74)		-	-1.26 (-2.28, -0.4)	-1.04 (-1.87, -0.21)	
0.34 (0.05, 0.74)	0.34 (-0.06, 0.77)	0.59 (0.17, 1.01)	1.18 (0.74, 1.66)	0.33 (-0.13, 0.8)	0.95 (0.46, 1.49)	**IVB+TA**	-0.08 (-0.45, 0.32)	-	
1.00 (-0.7, 1.34)	0.61 (-0.26, 0.98)	-0.79 (-1.38, 0.18)	**-0.49** **(-1.02, -0.6)**	-0.47 (-1.04, 0.18)	**-1.05** **(-1.52, -0.54)**		-	-	
0.19 (-0.37, 0.75)	0.19 (-0.43, 0.84)	0.44 (-0.01, 0.89)	1.06 (0.43, 1.66)	0.19 (-0.32, 0.69)	0.80 (0.17, 1.49)	**-0.15** **(-0.77, -0.01)**	**DEX+LP**	-	
-0.13 (-0.5, 0.21)	-0.54 (-0.97, 0.08)	0.62 (0.35, 0.9)	0.60 (-0.16, 1.03)	0.29 (-0.01, 0.6)	**-0.13** **(-0.51, -0.05)**	-0.27 (-0.7,1.63)		0.21 (-0.95, 1.56)	
**-0.83** **(-1.45, -0.3)**	**-0.83** **(-1.47, -0.25)**	-0.59 (-1.27, 0.03)	0.13 (-0.55, 0.5)	**-0.85** **(-1.54, -0.2)**	-0.23 (-0.66, 0.21)	**-1.18** **(-1.88, -0.53)**	**-1.03** **(-1.86, -0.28)**		**IVA**
0.71 (0.27, 1.11)	0.31 (-0.15, 0.74)	1.06 (0.98, 1.93)	1.45 (1.07, 1.77)	1.14 (0.64, 1.63)	**-0.72** **(-1.26, -0.14)**	0.33 (0.05, 0.6)	0.84 (-0.3, 1.38)	**IVR**	

*TA, intravitreal triamcinolone; IVB, intravitreal bevacizumab; LP, laser, macroscopic laser, grid laser and focal/grid laser; TA+LP, intrareal triamcinolone combined with laser; DEX, intravitreal dexamethasone; IVB+TA, intravitreal bevacizumab combined with triamcinolone; DEX+LP, intrareal dexamethasone combined with laser; IVR, intravitreal ranibizumab; IVA, intravitreal affiliation.

The blue represents for BCVA and green represents CMT. The bold values represent for P<0.05.

**Table 3:** SUCRA of network meta-analysis in multiple interventions at 3 months and 6 months.

	TA	IVB	LP	Placebo	TA+LP	DEX	IVB+TA	DEX+LP	IVR	IVA
BCVA-3 Month	64.47%	63.90%	39.39%	6.09%	66.36%	82.24%	94.09%	23.95%	9.22%	–
BCVA-6 Month	53.80%	75.28%	5.70%	7.63%	24.73%	98.82%	43.19%	53.52%	84.32%	–
CMT-3 Month	49.90%	16.00%	33.26%	0.11%	73.11%	44.37%	94.41%	88.86%	–	–
CMT-6 Month	47.91%	53.00%	6.70%	12.80%	36.09%	89.80%	–	68.80%	–	70.87%

*TA, intravitreal triamcinolone; IVB, intravitreal bevacizumab; LP, laser, macroscopic laser, grid laser and focal/grid laser; TA+LP, intrareal triamcinolone combined with laser; DEX, intravitreal dexamethasone; IVB+TA, intravitreal bevacizumab combined with triamcinolone; DEX+LP, intrareal dexamethasone combined with laser; IVR, intravitreal ranibizumab; IVA, intravitreal affiliation.

### 3.5 Mean change in central macular thickness of retina at 3 months

**Figure 6:**
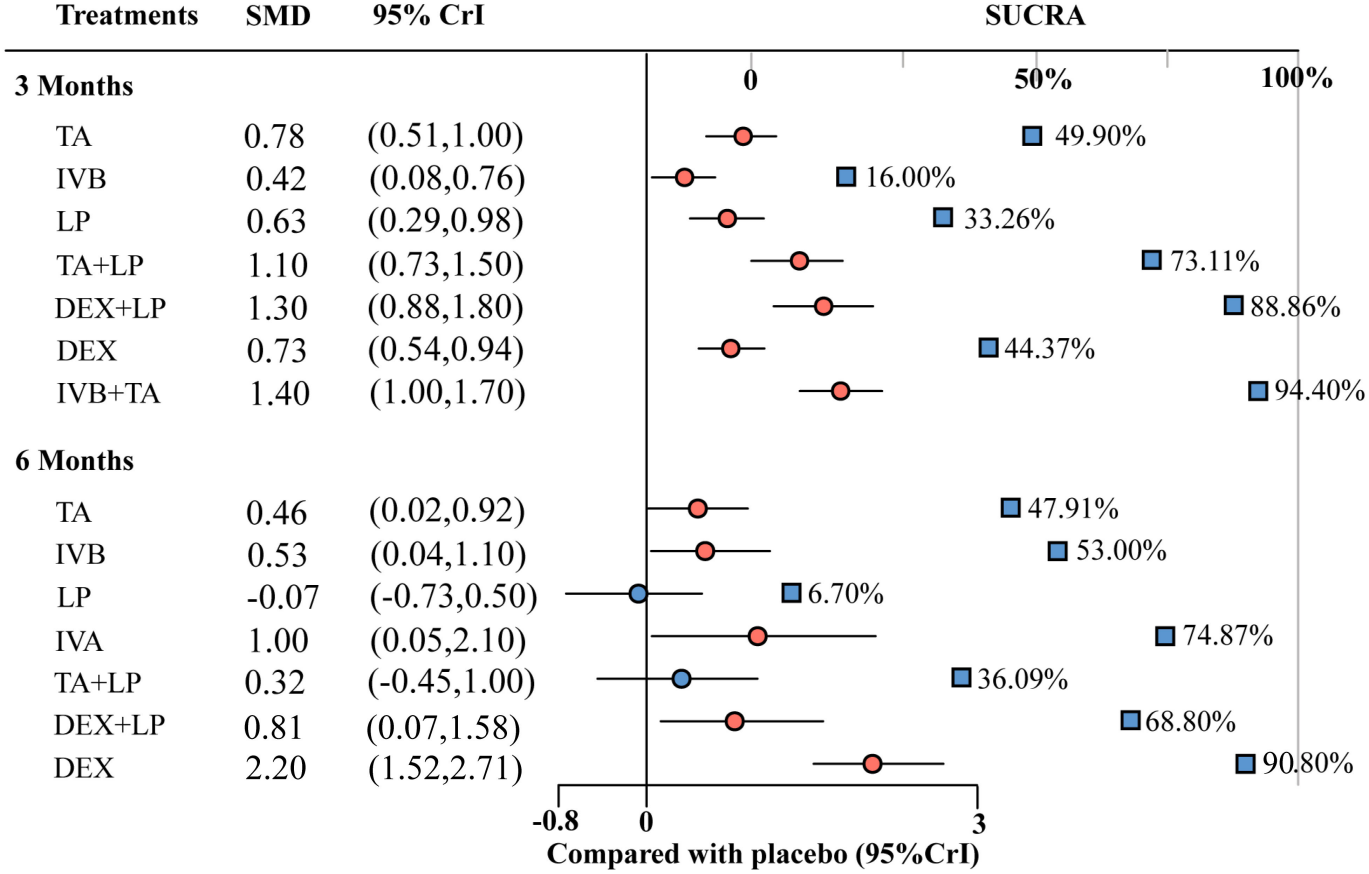
Forest plots for CMT at 3 months and 6 months. TA, intravitreal triamcinolone; IVB, intravitreal bevacizumab; LP, laser, macroscopic laser, grid laser and focal/grid laser; IVA, intravitreal affiliation; TA+LP, intrareal triamcinolone combined with laser; DEX, intravitreal dexamethasone; IVB+TA, intravitreal bevacizumab combined with triamcinolone; DEX+LP, intrareal dexamethasone combined with laser.

A total of 32 studies involving 4209 eyes were included in this study, with a total of 8 interventions, namely TA, IVB, LP, Placebo, TA+LP, DEX+LP, DEX and IVB+TA. A network meta-analysis was constructed (**Figure 4B**). In the forest plot of relative effect, DEX+LP and IVB+TA reduced the central macular thickness of patients with diabetic macular edema to approximately the same level, and their treatment effects were the best compared with other interventions. TA+LP was less effective in reducing macular edema. TA and DEX improved macular edema to a similar extent, and were only inferior to the first two levels of intervention; LP have similar effects, ranking fourth; IVB showed the least improvement in macular edema compared with placebo. Convergence tests were performed between interventions with head-to-head studies. The results of iterative convergence tests were expressed as PSRF values, and the closer the value to 1, the better the convergence. The PSRF of the network meta-analysis was 1, indicating good convergence. The values of each intervention compared with the placebo group were shown in **Table 2**. These results show that there was a significant difference in the reduction of central macular thickness after each intervention. The SUCRA values for each intervention were in **Table 3**. The SUCRA values and league table above showed that IVB+TA were the best interventions in reducing central macular thickness in patients with DME, followed by DEX+LP. IVB and Placebo had the worst results (**Figure 6**).

### 3.6 Mean change in best-corrected visual acuity at 6 months

A total of 9 interventions, TA, IVB, LP, Placebo, TA+LP, DEX+LP, DEX, IVB+TA and IVR, were included in this analysis, and 23 studies with a total of 3362 eyes were used to conduct a network meta-analysis and construct a network plot (**Figure 4C**). According to the forest plot of relative effect (placebo group as control), the effect of each intervention on improving patients’ vision can be divided into three levels: The effective schemes including DEX and IVR; The programs with moderate effect including IVB, TA, DEX+LP and IVB+TA; Less effective schemes including TA+LP and LP. Convergence tests were performed between interventions with head-to-head comparisons, and the results of iterative convergence tests were expressed as PSRF values, with values closer to 1 indicating better convergence. The PSRF of the network meta-analysis was 1, indicating good convergence. The values of interventions compared with the placebo group and the SUCRA values for each of the above interventions were shown in **Tables 2** and **3**, respectively. Combining the league table and SUCRA values, among these interventions, DEX were the best in improving best-corrected visual acuity at 6 months of treatment, LP and Placebo were relatively the worst (**Figure 5**).

### 3.7 Mean change in central macular thickness of retina at 6 months

A total of 25 studies involving 2858 eyes were included in this study, with a total of 8 interventions including TA, IVB, LP, Placebo, IVA, TA+LP, DEX+LP and DEX. A network meta-analysis was performed (**Figure 4D**). In the forest plot of relative effect constructed with the placebo group as control, at the 6-month treatment cycle, each intervention was better than the placebo group in reducing the central macular thickness of DME. The effect of DEX was the best, followed by IVA and DEX+LP, then followed by IVB and TA, while the effects of TA+LP and LP were not ideal. The PSRF of the mesh meta-analysis was 1, indicating good convergence. In the effect league table, the values of each intervention compared with the placebo group were in **Table 2**. The SUCRA values for each intervention were shown in **Table 3**. According to SUCRA value and league table analysis, DEX was the best in reducing central macular thickness and the effects of TA+LP and LP were relatively the worst (**Figure 6**).

### 3.8 Subgroup analysis of best-corrected visual acuity

**Figure 7:**
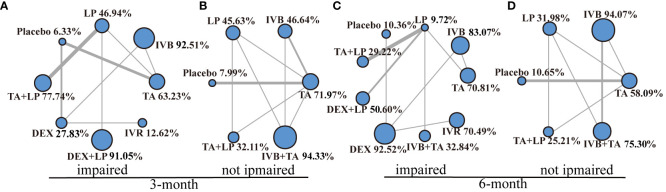
Network geometry for BCVA mean change from baseline. All populations of best-corrected visual acuity impaired and not impaired at 3-months follow-up [**(A, B)**] and 6-months follow-up [**(C, D)**]. BCVA, best-corrected visual acuity; CMT, central macular thickness. TA, intravitreal triamcinolone; IVB, intravitreal bevacizumab; LP, laser, macroscopic laser, grid laser and focal/grid laser; TA+LP, intrareal triamcinolone combined with laser; DEX, intravitreal dexamethasone; IVB+TA, intravitreal bevacizumab combined with triamcinolone; DEX+LP, intrareal dexamethasone combined with laser; IVR, intravitreal ranibizumab. Notes: Direct comparisons are represented by the black lines connecting the different interventions. Line width is proportional to the number of trials including every pair of interventions. The values of SUCRA are represented by the point size.

According to best-corrected visual acuity, diabetic macular edema was divided into two groups: best-corrected visual acuity impaired (<0.6) and best-corrected visual acuity not impaired (0.6~0.8). The network meta-analysis was performed separately (**Figure 7**). The values of each intervention were shown in the effect league table (**Supplementary 1**, **2**). At 3-month, IVB+TA had a best therapeutic effect on patients with intact vision. At 6-month, DEX was the best way to treat patients with impaired vision, while IVB is the best for patients without impaired vision.

### 3.9 Subgroup analysis of central macular thickness of retina

**Figure 8:**
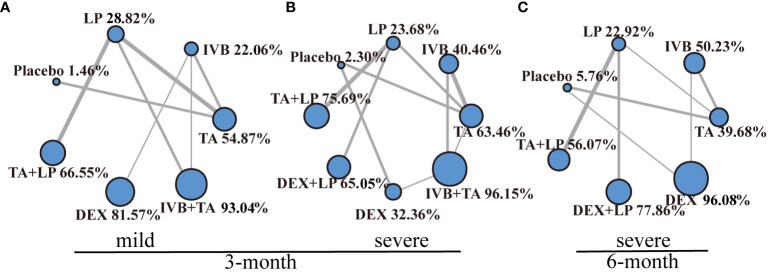
Network geometry for CMT mean change from baseline. All populations of mild macular edema and severe macular edema at 3-months follow-up [**(A, B)**]. All populations of severe macular edema at 6-months follow-up [**(C)**]. BCVA, best-corrected visual acuity; CMT, central macular thickness. macroscopic laser, grid laser and focal/grid laser; TA+LP, intrareal triamcinolone combined with laser; DEX, intravitreal dexamethasone; IVB+TA, intravitreal bevacizumab combined with triamcinolone; DEX+LP, intrareal dexamethasone combined with laser; IVR, intravitreal ranibizumab. Notes: Direct comparisons are represented by the black lines connecting the different interventions. Line width is proportional to the number of trials including every pair of interventions. The values of SUCRA are represented by the point size.

According to central macular thickness, diabetic macular edema was divided into two groups: mild macular edema (320~450μm) and severe macular edema (>450μm). The network meta-analysis was performed separately (**Figure 8**). The values of each intervention were shown in the effect league table (**Supplementary 3**, **4**). At 3-month, IVB+TA had a best therapeutic effect on patients no matter mild or severe macular edema. At 6-month, DEX was the best way to treat patients with severe macular edema, while the data of mild macular edema was unable to construct a network structure and be performed further analysis.

In addition, this study presents a risk table for the side effects associated with various interventions (**Supplementary Table S2**). It was observed that both TA and DEX have the potential to increase intraocular pressure. However, this adverse effect can be mitigated through the administration of intraocular pressure-lowering eye drops.

In terms of improving the best corrected visual acuity and reducing macular edema, the patients with diabetic macular edema who received IVB+TA had the best therapeutic effect at the 3-month follow-up. At the 6-month follow-up, DEX was the most effective treatment in improving central macular thickness. Glucocorticoids combined with laser therapy improved the efficacy at 3 and 6 months both, but LP alone had a poor effect. Furthermore, IVB+TA had a best therapeutic effect on patients with mild macular edema and best-corrected visual acuity not impaired at 3-month, while DEX was the best way to treat patients with severe macular edema and best-corrected visual acuity impaired.

## 4 Discussion

This network meta-analysis included 39 RCTs involving 5823 eyes. Through this study, we found that intravitreal injection of IVB + TA was the most beneficial for improving BCVA and reducing CMT compared with other treatments during the 3-month follow-up period. During the 6-month follow-up period, intravitreal DEX had the best effect in improving the BCVA and reducing the CMT.

We found that, in patients with diabetic macular edema at 3 months of follow-up, IVB+TA combined regimen improved the outcome of diabetic macular edema patients best, while TA, IVB alone regimen also achieved good clinical treatment effect. At the same time, glucocorticoids (TA or DEX) combined with LP achieved good results, too. At 6 months of follow-up, patients treated with the DEX regimen achieved the most significant improvement in visual quality and restoration of edematous macular anatomy. The combination of IVB+TA significantly improved vision and restored macular anatomy in patients with diabetic macular edema. This is consistent with the results of systematic review studies (50, 51). It might because the therapeutic effects of IVB + TA might be related to the antagonism of IVB against neovascularization and the antagonism of TA against macular local inflammation. The fundus retina of patients with diabetic retinopathy is in a state of ischemia and hypoxia due to local retinal microvascular damage. VEGF is highly concentrated in this area, which stimulates neovascularization around the lesion to compensate for ischemia and hypoxia. However, the neovascularization is fragile and easy to leak into the outer plexiform layer of the retina, resulting in macular edema (2, 52, 53). At the same time, bevacizumab, as a human monoclonal antibody targeting VEGF, inhibits angiogenesis by specifically binding to VEGF and blocking the signal transduction pathway of angiogenesis (54–56). In addition, inflammation plays an important role in the mechanism of DME. Chronic hyperglycemia in patients with DME induces oxidative stress and inflammation, resulting in retinal pericyte separation and structural changes in capillary tight junctions, causing blood-retinal barrier damage. Triamcinolone acetonide, a long-acting glucocorticoid, reduces inflammation by inducing the synthesis of anti-inflammatory cytokines and inhibiting the migration of inflammatory cells out of blood vessels. It stabilizes mast cells, reduces histamine release, shrinks capillary, and reduces vascular permeability (57, 58), thereby reducing inflammation-induced changes in the anatomy of the retina. Studies found that glucocorticoids (TA, DEX, etc.) can down-regulate the expression of various inflammatory factors including VEGF (57). Some studies showed that anti-VEGF was less effective in reducing foveal edema than other treatments, while glucocorticoid can eliminate macular foveal edema, and over time, the patient’s macular foveal regression effect is significant (59), thereby improving the central vision damage caused by retinal macular edema. On the other hand, previous meta-analyses did not compare the efficacy of TA alone with that of anti-VEGF alone, but we confirmed that the efficacy of TA alone was similar to that of anti-VEGF alone; The combination of IVB and TA was significantly superior to other anti-VEGF or glucocorticoid therapies during the 3-month follow-up period. TA and IVB, acting on separate pathways to combat inflammation and neovascularization, were less effective than the combination, but TA or IVB alone can also achieve significant efficacy and had a greater advantage over conventional LP. In addition, TA combined with LP had a significant therapeutic effect on central macular thickness in patients with DME, which was statistically different from LP. Therefore, clinicians should be aware of the benefits of TA+LP in improving visual acuity and reducing edema in the early treatment of DME. We also found that the effect of LP on macular edema was not significantly different from that of placebo at 3 and 6 months, suggesting that the role of LP in the treatment of DME should be re-evaluated; This differs from the findings of previous systematic reviews (60). It can be used as a supplement to combat macular edema, taking into account the economic burden of patients.

In this study, although there was a trend toward improvement in the efficacy of each intervention compared with the placebo group, the significance of the difference gradually decreased and the reasons are listed as below. On the one hand, the number of studies of the various interventions was insufficient, and the random effects model expanded the 95% confidence interval, leading to interventions other than TA were not statistically significant. At 3 and 6 months of follow-up, there was a trend of improvement in each treatment regimen compared with Placebo, but the difference was not statistically significant, which may be related to the lack of study size or the use of random effects model resulting in a wider 95%CI. Our results indicated that all regimens except placebo can improve the best corrected visual acuity and reduce macular thickness. In addition, the efficacy of DEX in improving the best corrected visual acuity of patients at 3 and 6 months of follow-up was comparable to that of placebo. Therefore, the effect of DEX on improving the best corrected visual acuity needs to be re-evaluated to eliminate the error caused by the risk of bias, and to clarify the actual impact of DEX on BCVA at 3 and 6 months, so as to provide a reliable basis for clinical selection of treatment regimens. On the other hand, most interventions showing statistical differences at 3 months and most interventions showing no statistical differences at 6 months. The reason may be that the efficacy of the intervention diminished at 6 months, with only DEX remaining effective.

This might be related to the duration of DEX action and the progression of regression of macular edema in the central retina. Compared with triamcinolone acetonide, DEX implants have a delayed onset of action, slower drug release, and longer duration of action after intravitreal injection (61). DEX exhibits a biphasic sustained release with high concentrations for the first 2 months and low concentrations for several months, resulting in a longer period of modulation of VEGF expression and anti-inflammatory effects in the vitreous (62, 63). We also found that intravitreal injection of DEX also provided significant improvement in patients treated with LP within 3 months before and after injection, regardless of the order of treatment of LP and DEX. In our study of 6-month CMT, although no study of IVB combined with TA was included, it can still provide a reference for clinical treatment. In addition, anti-VEGF treatment resistance may affect the results. Some studies showed that patients with certain types of diabetic macular edema were resistant to anti-VEGF drug therapy and did not achieve significant visual and anatomical improvement (64). Therefore, future research may focus on these patients who are resistant to anti-VEGF drugs during clinical treatment.

This network meta-analysis studied the common drugs used in the treatment of DME in clinical practice, including anti-VEGF drugs, glucocorticoids and laser. Unlike previous network meta-analyses, which only paid attention to the study of anti-VEGF drugs, and ignored the important effect of glucocorticoids, such as TA and DEX, in improving the visual acuity and central macular edema thickness, our study evaluated the improvement of visual acuity and macular structure recovery in patients with glucocorticoids in 3-month and 6-month short cycle treatment, thus providing more references for clinicians to choose. In addition, we combined the data of single or combination therapy and found that DEX combined with LP can also achieve satisfactory clinical efficacy. Previous meta-analyses did not assess the effect of DEX in combination with LP, focusing only on monotherapy (65), but our research made up for this deficiency.

Our study also had some limitations. First, a total of 39 studies were included in the network meta-analysis, but it was not assessed whether the number of included studies met the requirements for testing publication bias. In addition, the central macular thickness of the retina did not form a closed loop at the 6-month follow-up, and the inconsistency test could not be completed. Second, there were some contradictory phenomena in our study. For example, LP was found to be less effective than placebo in reducing macular edema at 3 months. Besides, the results of LP and TA+LP in improving foveal edema at 6 months were implausible and inconsistent with the efficacy outcomes of drugs in clinical practice. It was speculated that there may be two reasons. On the one hand, the number and sample size of the research projects were small. On the other hand, the included studies on the single treatment of some measures may have large heterogeneity, which may impair the reliability of the network meta-analysis results of this group. Third, we did not pay attention to the type of diabetes, the stage of diabetic retinopathy, and the effects of other syndrome. Whether these conditions can change the outcome of medical treatment of diabetic macular edema needs further study. Fourth, this study only focused on the efficacy of glucocorticoid alone, and the evaluation of the efficacy and safety of glucocorticoid combined with anti-VEGF treatment regimen is not perfect. Side effects such as endophthalmitis caused by hormone therapy were not mentioned in all included studies, so the safety evaluation of adverse reactions caused by drugs should be paid continuous attention. In addition, the number of studies on DEX+LP is insufficient, and there are limitations in evaluating its effectiveness. Further studies are needed to address these gaps and provide a more comprehensive understanding of its therapeutic potential. Therefore, in order to more accurately analyze the efficacy and safety of drug treatment of diabetic macular edema, more multi-center, multi-ethnic, multi-regional, high-quality prospective randomized controlled trials are required.

## 5 Conclusion

This study showed that intravitreal injection of IVB+TA was most beneficial in improving best-corrected visual acuity and reducing the thickness of macular edema in the center of the retina compared with other treatments during the 3-month follow-up period. Intravitreal injection of DEX was the most effective in improving best-corrected visual acuity and reducing central macular edema thickness over a 6-month follow-up period, especially the patients with severe macular edema and visual acuity impaired. Given the limitations of this study, the results need to be interpreted with caution, and more well-designed RCTs are warranted in the future.

## Data availability statement

The original contributions presented in the study are included in the article/**Supplementary Material**. Further inquiries can be directed to the corresponding author.

## Author contributions

Z’AC: Writing – original draft, Software, Methodology, Investigation, Formal Analysis, Data curation, Conceptualization. XL: Writing – review & editing, Visualization, Supervision, Project administration, Investigation, Conceptualization.
