# Supplementary material for: Comparing the efficacy of glucocorticoids and anti-VEGF in treating diabetic macular edema: systematic review and comprehensive analysis
Source: Front Endocrinol (Lausanne). 2024 Mar 22;15:1342530. doi: 10.3389/fendo.2024.1342530 (PMC10995385; doi:10.3389/fendo.2024.1342530)
Supplement: Supplementary file 1 [file DataSheet_1.docx]

| **TA** | 0.17  (-0.27, 0.65) | 0.19  (-0.25, 0.65) | 0.61  (0.11, 1.09) | 0.30  (-0.22, 0.86) | - | - | -0.30  (-0.73, 0.16) | |
| --- | --- | --- | --- | --- | --- | --- | --- | --- |
| 0.25  (-0.37, 0.85) | **IVB** | 0.01  (-0.6, 0.61) | 0.43  (-0.26, 1.09) | 0.13  (-0.56, 0.81) | - | - | **-0.47**  **(-0.96, 0)** | |
| -0.18  (-0.8, 0.45) | -0.43  (-1.32, 0.47) | **LP** | 0.42  (-0.25, 1.07) | 0.12  (-0.4, 0.63) | - |  | **-0.48**  **(-0.98, 0)** | |
| **-0.94**  **(-1.32, -0.55)** | **-1.2**  **(-1.79, -0.56)** | **-0.76**  **(-1.5, -0.02)** | **Placebo** | -0.30  (-1.03, 0.44) | - | -- | **-0.90**  **(-1.56, -0.23)** | |
| 0.17  (-0.5, 0.9) | -0.08  (-1, 0.9) | 0.36  (0.08, 0.68) | 1.12  (0.35, 1.93) | **TA+LP** | **-** | - | **-0.60**  **(-1.24, -0.04)** | |
| **-0.64**  **(-1.16, -0.17)** | **-0.89**  **(-1.49, -0.3)** | -0.45  (-1.28, 0.32) | 0.31  (-0.1, 0.61) | **-0.81**  **(-1.72, -0.01)** | **DEX** | **-** | - | |
| 0.26  (-0.55, 1.09) | 0.01  (-1, 1.05) | 0.44  (-0.08, 0.97) | 1.20  (0.3, 2.11) | 0.09  (-0.55, 0.67) | 0.89  (-0.03, 1.88) | **DEX+LP** | **-** | |
| **-0.87**  **(-1.61, -0.2)** | **-1.12**  **(-1.9, -0.35)** | -0.68  (-1.67, 0.23) | 0.08  (-0.6, 0.65) | **-1.04**  **(-2.1, -0.1)** | -0.23  (-0.75, 0.28) | **-1.12**  **(-2.25, -0.08)** |  | **IVB+TA** |
|  |  |  |  |  |  |  | **IVR** |  |

Network meta-analysis results in BCVA impaired (lower part) and not impaired (upper part) at 3 months.
