# Supplementary material for: Comparing the efficacy of glucocorticoids and anti-VEGF in treating diabetic macular edema: systematic review and comprehensive analysis
Source: Front Endocrinol (Lausanne). 2024 Mar 22;15:1342530. doi: 10.3389/fendo.2024.1342530 (PMC10995385; doi:10.3389/fendo.2024.1342530)
Supplement: Supplementary file 2 [file DataSheet_2.docx]

| **TA** | -0.58  (-1.3, 0.15) | 0.27  (0, 0.78) | 0.54  (0, 1.05) | 0.35  (-0.27, 1.02) | - | - | -0.25  (-0.93, 0.55) | - |
| --- | --- | --- | --- | --- | --- | --- | --- | --- |
| 0.19  (-0.67, 1.05) | **IVB** | 0.85  (0.15, 1.62) | 1.04  (0.2,1.85) | 0.92  (0.08, 1.83) | - | - | 0.32  (-0.19, 0.95) | - |
| **-1.22**  **(-2.19, -0.25)** | **-1.41**  **(-2.71, -0.11)** | **LP** | 0.29  (0.03,1.0) | 0.07  (-0.55, 0.69) | - | - | **-0.53**  **(-1.14, -0.14)** | **-** |
| **-1.4**  **(-2.87,-0.08)** | **-1.59**  **(-2.78, -0.4)** | **-0.18**  **(-0.96, -0.09)** | **Placebo** | -0.19 (-1, 0.7) | -- | - | -0.79  (-1.67, 0.22) | - |
| -0.91  (-1.93, 0.16) | -1.1  (-2.44, 0.29) | 0.31  (-0.05, 0.73) | 0.49  (-1.3, 2.33) | **TA+LP** |  | - | **-0.6**  **(-1.43, -0.28)** | **-** |
| -0.44  (-1.54, 0.72) | -0.63  (-2.03, 0.82) | 0.77  (0.23, 1.39) | 0.96  (-0.87, 2.85) | 0.47  (-0.23, 1.18) | **DEX+LP** | **-** |  |  |
| 0.42  (-0.85, 1.7) | 0.23 (0.06,0.71) | 1.64  (0.03, 3.25) | 1.82  (1.06, 2.58) | 1.33  (-0.37, 2.96) | 0.86  (0.88, 2.54) | **DEX** |  | - |
| -0.83  (-2.08, 0.43) | -1.02  (-2.55, 0.52) | 0.4  (-0.4, 1.19) | 0.58  (-1.36, 2.51) | 0.09  (-0.83, 0.95) | -0.38  (-1.4, 0.57) | **-1.24**  **(-3.03, -0.56)** | **IVB+TA** | **-** |
| 0.09  (-1.39, 1.56) | -0.1  (-1.3, 1.08) | 1.31  (-0.47, 3.08) | 1.49  (0.4, 2.57) | 1  (0.86, 2.81) | 0.54  (-1.37, 2.36) | -0.33  (-1.1, 0.43) | 0.91  (-1.05, 2.87) | **IVR** |

Network meta-analysis results in BCVA impaired (lower part) and not impaired (upper part) at 6 months.
