# Supplementary material for: Comparing the efficacy of glucocorticoids and anti-VEGF in treating diabetic macular edema: systematic review and comprehensive analysis
Source: Front Endocrinol (Lausanne). 2024 Mar 22;15:1342530. doi: 10.3389/fendo.2024.1342530 (PMC10995385; doi:10.3389/fendo.2024.1342530)
Supplement: Supplementary file 3 [file DataSheet_3.docx]

Network meta-analysis results in CMT with mild macular edema (lower part) and severe macular edema (upper part) at 3 months.

| **TA** | -0.33  (-0.91, 0.23) | -0.61  (-1.41, 0.11) | **-1.24**  **(-2, -0.57)** | 0.27  (-0.74, 1.21) | 0.1  (-0.96, 1.1) | -0.51  (-1.4, 0.31) | 0.82  (0.16, 1.49) |
| --- | --- | --- | --- | --- | --- | --- | --- |
| 0.32  (-0.08, 0.72) | **IVB** | -0.28  (-1.25, 0.64) | **-0.91**  **(-1.81, -0.07)** | 0.6  (-0.55, 1.7) | 0.43  (-0.77, 1.58) | -0.18  (-1.16, 0.75) | 1.15  (0.53, 1.8) |
| 0.2  (-0.07, 0.46) | -0.12  (-0.54, 0.3) | **LP** | -0.63  (-1.68, 0.4) | 0.88  (0.28, 1.49) | 0.71  (0.01, 1.42) | 0.1  (-1.03, 1.25) | 1.43  (0.46, 2.49) |
| **0.75**  **(0.34, 1.14)** | 0.43  (-0.15, 0.99) | **0.55**  **(0.07, 1.03)** | **Placebo** | 1.51  (0.32, 2.73) | 1.34  (0.1, 2.6) | 0.73  (0.11, 1.38) | 2.07  (1.23, 2.97) |
| -0.11  (-0.46, 0.23) | -0.43  (-0.91, 0.04) | **-0.31**  **(-0.53, -0.09)** | **-0.87**  **(-1.38, -0.32)** | **TA+LP** | -0.17  (-1.1, 0.76) | -0.78  (-2.07, 0.52) | 0.55  (-0.59, 1.77) |
|  |  |  |  |  | **DEX+LP** | -0.61  (-1.94, 0.73) | 0.72  (-0.47, 1.99) |
| -0.4  (-1.19, 0.37) | **-0.71**  **(-1.38, -0.05)** | -0.59  (-1.39, 0.19) | **-1.14**  **(-2.04, -0.28)** | -0.28  (-1.09, 0.53) |  | **DEX** | 1.33  (0.45, 2.28) |
| **-0.48**  **(-0.86, -0.1)** | **-0.8**  **(-1.21, -0.4)** | **-0.68**  **(-0.99, -0.36)** | **-1.23**  **(-1.77, -0.68)** | -0.37  (-0.75, 0.02) |  | **-0.43**  **(-0.85, 0.07)** | **IVB+TA** |
