# Supplementary material for: Comparing the efficacy of glucocorticoids and anti-VEGF in treating diabetic macular edema: systematic review and comprehensive analysis
Source: Front Endocrinol (Lausanne). 2024 Mar 22;15:1342530. doi: 10.3389/fendo.2024.1342530 (PMC10995385; doi:10.3389/fendo.2024.1342530)
Supplement: Supplementary file 4 [file DataSheet_4.docx]

Network meta-analysis results in CMT with severe macular edema at 6 months.

| **TA** |  | | | | | |
| --- | --- | --- | --- | --- | --- | --- |
| -0.13  (-0.76, 0.44) | **IVB** |  | | | | |
| 0.21  (-0.71, 1.12) | 0.34  (-0.73, 1.47) | **LP** |  | | | |
| 0.6  (0.01, 1.2) | 0.73  (0.03, 1.51) | 0.39  (-0.7, 1.49) | **Placebo** |  | | |
| -0.25  (-1.32, 0.8) | -0.12  (-1.32, 1.12) | -0.47  (-1, 0.08) | -0.85  (-2.1, 0.35) | **TA+LP** |  | |
| -0.66  (-1.77, 0.45) | -0.53  (-1.76, 0.76) | **-0.87**  **(-1.5, -0.24)** | **-1.26**  **(-2.52, -0.01)** | -0.4  (-1.24, 0.42) | **DEX+LP** |  |
| **-1.39**  **(-2.24, -0.69)** | **-1.26**  **(-2.03, -0.56)** | **-1.61**  **(-2.86, -0.47)** | **-1.99**  **(-2.76, -1.37)** | -1.14  (-2.51, 0.13) | **-1.73**  **(-2.14, -0.56)** | **DEX** |
