# Supplementary material for: Comparing the efficacy of glucocorticoids and anti-VEGF in treating diabetic macular edema: systematic review and comprehensive analysis
Source: Front Endocrinol (Lausanne). 2024 Mar 22;15:1342530. doi: 10.3389/fendo.2024.1342530 (PMC10995385; doi:10.3389/fendo.2024.1342530)
Supplement: Supplementary file 5 [file Table_1.docx]

**Table S1** Basic information of randomized controlled trials included in the network meta-analysis.

| Study | Intervention | Treatment dose(mg) | Frequency | Time(interval/total time) |
| --- | --- | --- | --- | --- |
| Audren 2006 | TA | 4 | 3 | 4week/24week |
|  | Placebo | 4 | NA | 0week/24week |
| Aydin 2009 | TA+LP | 4+standard laser | 2 | 3mon/6mon |
|  | TA | 4 | NA | 0week /6mon |
| Azad 2012 | TA | 4 | 1 | 0mon/6mon |
|  | IVB | 1.25 | 1 | 0mon/6mon |
| Bhayana 2015 | TA | 20 | 2 | 3mon/6mon |
|  | IVB | 1.25 | NA | NA/6mon |
| Boyer 2014 | DEX | 0.7 | 1 | 0mon/6mon |
|  | Placebo | 0.7 | 1 | 0mon/6mon |
| Callanan 2013 | DEX+LP | 0.7 | 2 | 0,4mon/6mon |
|  | LP | NA | 2 | 0,4mon/6mon |
| Callanan 2017 | DEX | 0.7 | 2 | 5mon/10mon |
|  | IVR | 0.5 | 2 | 5mon/10mon |
| Comet 2021 | DEX | 0.7 | 2 | 3mon/6mon |
|  | IVA | 2 | 2 | 3mon/6mon |
| Danis 2016 | DEX | 0.7 | 2 | 3mon/3mon |
|  | Placebo | 0.7 | NA | NA/3mon |
| Dehghan 2008 | TA | 4 | 3 | 2mon/4mon |
|  | Placebo | 4 | 3 | 2mon/4mon |
| Elman 2010 | TA+LP | 4+ NA | 2 | 4week/6mon |
|  | LP | NA | 2 | 4week/6mon |
| Emily 2007 | TA | 4 | 2 | 0,4mon/6mon |
|  | LP | NA | NA | NA/6mon |
| Emily 2007' | TA+LP | 4/ NA | 2 | 0,4mon/6mon |
|  | LP | NA | 2 | 0,4mon/6mon |
| Faghihi 2008 | IVB+TA | 1.25+2.0 | 2 | 6week/16week |
|  | TA | 1.25 | 2 | 6week/16week |
| Fazel 2023 | IVB+TA | 1.25+4 | 2 | 2mon/12week |
|  | IVB | 1.25 | 2 | 2mon/12week |
| Gao 2022 | DEX+LP | 0.7 | 3 | 1mon/6mon |
|  | LP | 0.5 | 3 | 1mon/6mon |
| Gil 2011 | TA | 4 | NA | 0mon/6mon |
|  | LP | standard laser | NA | 0mon/6mon |
| Gillies 2010 | TA+LP | 4+ NA | 2 | 0,3mon/6mon |
|  | LP | NA | 2 | 0,3mon/6mon |
| Gillies 2014 | DEX | 0.7 | 2 | 0,16week/6mon |
|  | IVB | 1.25 | 6 | 0, 4,8,12,16,20week/6mon |
| Heng 2016 | DEX+LP | NA | 2 | 16week/56week |
|  | LP | NA | NA | NA/56week |
| Isaac 2012 | TA | 4 | 1 | 0week/24week |
|  | IVB | 1.25 | 1 | 0week/24week |
| Jonas 2004 | TA | 25 | 2 | 3mon/6mon |
|  | Placebo | 25 | NA | NA/6mon |
| Kriechbaum 2014 | TA | 8 | 3 | 4,8week/12mon |
|  | IVB | 2.5 | 3 | 4,8week/12mon |
| Lam 2007 | TA+LP | 4+standard laser | 2 | 4mon/6mon |
|  | LP | standard laser | NA | NA/6mon |
| Lam 2007' | TA | 4 | 2 | 4mon/6mon |
|  | LP | standard laser | NA | NA/6mon |
| Larsson 2009 | TA | 4 | NA | NA/3mon |
|  | Placebo | 4 | NA | NA/3mon |
| Lee 2009 | TA+LP | 4+standard laser | 1 | 0mon/6mon |
|  | LP | standard laser | NA | NA |
| Li 2014 | TA+LP | 20+standard laser | 2 | 3mon/6mon |
|  | LP | standard laser | 4 | every week/6mon |
| Maia Jr 2009 | TA | 4 | 1 | 0mon/6mon |
|  | LP | NA | 3 | 1,2,3week/6mon |
| Marey 2011 | TA | 4 | 1 | 0week/12week |
|  | IVB+TA | 4+1.25 | 1 | 0week/12week |
| Marey 2011' | TA | 4 | 1 | 0week/12week |
|  | IVB | 1.25 | 1 | 0week/12week |
| Marey 2011'' | IVB+TA | 1.25+4 | 1 | 0week/12week |
|  | IVB | 1.25 | 1 | 0week/12week |
| Massin 2004 | TA | 4 | 1 | 0week/24week |
|  | Placebo | NA | 1 | 0week/24week |
| Meyer 2022 | DEX | 0.7 | 2 | 0,3mon/6mon |
|  | IVB | 1.25 | 5 | 0,1,2,4,5mon/6mon |
| Ockrim 2008 | TA | 4 | 2 | 4mon/12mon |
|  | LP | NA | NA | NA |
| Ogura 2019 | TA | 20 | 1 | 0week/12week |
|  | LP | NA | NA | NA |
| Ozsaygili 2020 | DEX | 0.7 | 1 | 3mon/6mon |
|  | IVA | 2 | 3 | 4week/6mon |
| Soheilian 2007 | IVB+TA | 1.25+2 | 1 | 0week/12week |
|  | LP | 1.25 | 1 | 0week/12week |
| Soheilian 2009 | IVB+TA | 1.25+2 | 3 | 12week/24week |
|  | IVB | 1.25 | 3 | 12week/24week |
| Soheilian 2009' | IVB+TA | 1.25+2 | 3 | 12week/24week |
|  | LP | NA | 3 | 12week/24week |
| Stefansson 2023 | DEX | 1.35 | NA | 0week/12week |
|  | LP | NA | NA | 0week/12week |
| Sutter 2004 | TA | 0.1 | 1 | 0mon/3mon |
|  | Pacebo | 0.1 | NA | 0mon/3mon |
| Wei 2021 | DEX+LP | 0.7 | 2 | 5mon/6mon |
|  | LP | NA | 3 | 3mon/6mon |
| Yaseri 2014 | IVB+TA | 1.25+2 | 3 | 12week/6mon |
|  | IVB | 1.25 | 3 | 12week/6mon |
| Yaseri 2014' | IVB+TA | 1.25+2 | 3 | 12week/6mon |
|  | LP | NA | 3 | 12week/6mon |

* TA, intravitreal triamcinolone; IVB, intravitreal bevacizumab; LP, laser, macroscopic laser, grid laser and focal/grid laser; TA+LP, intrareal triamcinolone combined with laser; DEX, intravitreal dexamethasone; IVB+TA, intravitreal bevacizumab combined with triamcinolone; DEX+LP, intrareal dexamethasone combined with laser; IVR, intravitreal ranibizumab; IVA, intravitreal affiliation; standard laser, 4/50–200µm,100–220mW,0.1–0.2s.
