# Supplementary material for: Comparing the efficacy of glucocorticoids and anti-VEGF in treating diabetic macular edema: systematic review and comprehensive analysis
Source: Front Endocrinol (Lausanne). 2024 Mar 22;15:1342530. doi: 10.3389/fendo.2024.1342530 (PMC10995385; doi:10.3389/fendo.2024.1342530)
Supplement: Supplementary file 6 [file Table_2.docx]

**Table S2** The side effects of various treatments included in the network meta-analysis.

| Study | Intervention | Eye | Ocular hypertension | Cataract | Conjunctival hemorrhage |
| --- | --- | --- | --- | --- | --- |
| Audren 2006 | TA | 17 | 0.00% | - | - |
|  | Placebo | 17 | 0.00% | - | - |
| Aydin 2009 | TA+LP | 24 | 0.00% | - | - |
|  | TA | 25 | 0.00% | - | - |
| Azad 2012 | TA | 20 | 50.00% | 50.00% | - |
|  | IVB | 20 | 0.00% | 20.00% | - |
| Bhayana 2015 | TA | 15 | 11.80% | - | - |
|  | IVB | 15 | 0.00% | - | - |
| Boyer 2014 | DEX | 688 | 32.00% | - | - |
|  | Placebo | 360 | 4.30% | - | - |
| Callanan 2013 | DEX+LP | 180 | - | - | - |
|  | LP | 73 | - | - | - |
| Callanan 2017 | DEX | 152 | 36.50% | 9.40% | 18.20% |
|  | IVR | 211 | 2.20% | 0.00% | 12.10% |
| Comet 2021 | DEX | 20 | 0.00% | 0.00% | 0.00% |
|  | IVA | 21 | 0.00% | 0.00% | 0.00% |
| Danis 2016 | DEX | 731 | NA | - | - |
|  | Placebo | 303 | NA | - | - |
| Dehghan 2008 | TA | 44 | 7.10% | - | - |
|  | Placebo | 44 | 0.00% | - | - |
| Elman 2010 | TA+LP | 215 | 2.50% | 15.00% | - |
|  | LP | 264 | 1.00% | 6.00% | - |
| Emily 2007 | TA | 41 | 17.07% | - | - |
|  | LP | 41 | 2.43% | - | - |
| Emily 2007' | TA+LP | 44 | 4.54% | - | - |
|  | LP | 41 | 2.43% | - | - |
| Faghihi 2008 | IVB+TA | 42 | - | - | - |
|  | TA | 41 | - | - | - |
| Fazel 2023 | IVB+TA | 30 | - | - | - |
|  | IVB | 28 | - | - | - |
| Gao 2022 | DEX+LP | 18 | 13.30% | - | - |
|  | LP | 18 | 5.56% | - | - |
| Gil 2011 | TA | 11 | 0.00% | 0.00% | 0.00% |
|  | LP | 10 | 0.00% | 0.00% | 0.00% |
| Gillies 2010 | TA+LP | 42 | 3.20% | - | - |
|  | LP | 42 | 1.50% | - | - |
| Gillies 2014 | DEX | 44 | NA | 6.50% | - |
|  | IVB | 44 | NA | 2.40% | - |
| Heng 2016 | DEX+LP | 40 | 0.00% | 30.00% | - |
|  | LP | 40 | 0.00% | 14.80% | - |
| Isaac 2012 | TA | 11 | 27.30% | 0.00% | - |
|  | IVB | 11 | 0.00% | 0.00% | - |
| Jonas 2004 | TA | 25 | 5.20% | - | - |
|  | Placebo | 25 | 0.00% | - | - |
| Kriechbaum 2014 | TA | 15 | NA | 0.00% | 0.00% |
|  | IVB | 15 | NA | 0.00% | 0.00% |
| Lam 2007 | TA+LP | 38 | NA | - | - |
|  | LP | 35 | NA | - | - |
| Lam 2007' | TA | 40 | NA | 17.00% | - |
|  | LP | 35 | NA | - | - |
| Larsson 2009 | TA | 16 | 4.60% | - | - |
|  | Placebo | 16 | 2.10% | - | - |
| Lee 2009 | TA+LP | 30 | 6.10% | - | - |
|  | LP | 30 | 3.60% | - | - |
| Li 2014 | TA+LP | 32 | NA | - | - |
|  | LP | 32 | NA | - | - |
| Maia Jr 2009 | TA | 22 | 10.50% | 1.50% | - |
|  | LP | 22 | 4.71% | 2.10% | - |
| Marey 2011 | TA | 30 | - | - | - |
|  | IVB+TA | 30 | - | - | - |
| Marey 2011' | TA | 30 | - | - | - |
|  | IVB | 30 | - | - | - |
| Marey 2011'' | IVB+TA | 30 | - | - | - |
|  | IVB | 30 | - | - | - |
| Massin 2004 | TA | 12 | 6.40% | 1.20% | - |
|  | Placebo | 12 | 3.50% | 0.00% | - |
| Meyer 2022 | DEX | 26 | NA | - | - |
|  | IVB | 26 | NA | - | - |
| Ockrim 2008 | TA | 44 | - | - | - |
|  | LP | 44 | - | - | - |
| Ogura 2019 | TA | 45 | 9.40% | 3.30% | 3.00% |
|  | LP | 44 | 3.30% | 10.00% | 0.00% |
| Ozsaygili 2020 | DEX | 48 | 5.00% | - | - |
|  | IVA | 50 | 6.00% | - | - |
| Soheilian 2007 | IVB+TA | 33 | 9.10% | - | - |
|  | LP | 33 | 0.00% | - | - |
| Soheilian 2009 | IVB+TA | 50 | 10.50% | - | - |
|  | IVB | 50 | 8.00% | - | - |
| Soheilian 2009' | IVB+TA | 50 | 10.50% | - | - |
|  | LP | 50 | 3.00% | - | - |
| Stefansson 2023 | DEX | 72 | 3.00% | 2.00% | - |
|  | LP | 72 | 2.20% | 2.20% | - |
| Sutter 2004 | TA | 32 | NA | NA | - |
|  | Placebo | 33 | NA | NA | - |
| Wei 2021 | DEX+LP | 141 | 33.80% | 15.20% | - |
|  | LP | 131 | 4.70% | 6.20% | - |
| Yaseri 2014 | IVB+TA | 10 | 7.30% | - | - |
|  | IVB | 9 | 0.00% | - | - |
| Yaseri 2014' | IVB+TA | 11 | 7.30% | - | - |
|  | LP | 11 | 0.00% | - | - |

* TA, intravitreal triamcinolone; IVB, intravitreal bevacizumab;LP, laser, macroscopic laser, grid laser and focal/grid laser;TA+LP, intrareal triamcinolone combined with laser; dex, intravitreal dexamethasone; IVB+TA, intravitreal bevacizumab combined with triamcinolone; DEX+LP, intrareal dexamethasone combined with laser; IVR, intravitreal ranibizumab;IVA, intravitreal affiliation.
